# Capturing High-Frequency Harmonic Signatures for NILM: Building a Dataset for Load Disaggregation

**DOI:** 10.3390/s25154601

**Published:** 2025-07-25

**Authors:** Farid Dinar, Sébastien Paris, Éric Busvelle

**Affiliations:** 1 Laboratoire d’Informatique et des Systèmes (LIS), Unité Mixte de Recherche, Centre National de la Recherche Scientifique (UMR, CNRS) 7020, Université de Toulon, Aix Marseille Université, 83130 La Garde, Francebusvelle@univ-tln.fr (É.B.); 2 Electrical Engineering and Computer Science Department, Institut Universitaire de Technologie (IUT) de Toulon, Av. de l’Université, 83130 La Garde, France

**Keywords:** electrical measurements based on high-frequency signals, non-intrusive load monitoring (NILM), energy disaggregation, embedded systems, harmonic analysis, machine learning, smart energy systems

## Abstract

Advanced Non-Intrusive Load Monitoring (NILM) research is important to help reduce energy consumption. Very-low-frequency approaches have traditionally faced challenges in separating appliance uses due to low discriminative information. The richer signatures available in high-frequency electrical data include many harmonic orders that have the potential to advance disaggregation. This has been explored to some extent, but not comprehensively due to a lack of an appropriate public dataset. This paper presents the development of a cost-effective energy monitoring system scalable for multiple entries while producing detailed measurements. We will detail our approach to creating a NILM dataset comprising both aggregate loads and individual appliance measurements, all while ensuring that the dataset is reproducible and accessible. Ultimately, the dataset can be used to validate NILM, and we show through the use of machine learning techniques that high-frequency features improve disaggregation accuracy when compared with traditional methods. This work addresses a critical gap in NILM research by detailing the design and implementation of a data acquisition system capable of generating rich and structured datasets that support precise energy consumption analysis and prepare the essential materials for advanced, real-time energy disaggregation and smart energy management applications.

## 1. Introduction

The increasing awareness of energy efficiency and sustainable resource management has accelerated the development of technologies related to smart grids and intelligent monitoring energy systems. In this landscape, Non-Intrusive Load Monitoring (NILM) has recently emerged as a method of disaggregation of total energy consumption for residences into individual appliance levels. Traditional performance monitoring for known appliances typically requires dedicated sensors for each appliance, making installation and data collection complicated and very expensive. NILM avoids many of these challenges by estimating an appliance’s specific consumption based on one aggregate measurement point. This simple but effective methodology can benefit consumers, as well as utility supply providers. It can provide consumers with more detailed, appliance-based energy audits and allow them to easily identify power consumption anomalies, and it also supports appliance-based personal energy management while limiting the utility hardware costs associated with deployment. However, NILM has a number of application challenges; the most notable is the challenge of appliance identification in real-world environments where multiple devices operate simultaneously, usually with overlapping load signatures.

A crucial factor limiting NILM performance is the quality and resolution of power consumption data. Conventional NILM approaches relying on low-resolution measurements of active and reactive power often face significant challenges in distinguishing between appliances [1]. This limitation has driven interest in high-frequency energy monitoring, as detailed data can reveal richer electrical characteristics across different operational states. Prior research [2,3] has shown that incorporating high-frequency features, namely, harmonic components, can significantly improve classification accuracy for certain appliance types [4]. However, the expanded development of such datasets has been prevented by the high cost of precision measurement equipment, difficulties in long-term deployment, and the computational charge associated with processing and storing vast amounts of high-resolution data. One of the ongoing problems in NILM applications is high-frequency feature extraction [5]. One of the biggest limitations in this regard is the need to maintain a high sampling frequency in order to capture transient events, which can be challenging to achieve, especially without developing an application-specific sensing device [1]. In this study, we demonstrate the design, development, and testing of a scalable and cost-effective system for capturing and collecting electrical data for NILM applications. The system captures a certain set of electrical properties, including voltage, current, active power, apparent power, power factor, and current harmonic amplitudes up to order 32, at a high-frequency sampling. Using four dedicated nodes (sensors) for measuring electrical quantities, this system enables accurate monitoring at both aggregated and individual appliance levels, providing high-quality ground truth data in real time. Unlike many publicly available datasets, the data presented in this work provide the ability to obtain more resolution with more complex structures, with the potential to produce major contributions to advanced load disaggregation methods.

Thus, the principal contributions made in this study are the development of scalable, affordable systems destined for NILM applications, with available commercial hardware, making advanced energy monitoring available to a larger audience. Additionally, a dataset was created that includes high-frequency-based measurements among a diverse set of appliances, providing useful information on electric consumption in many different scenarios. The dataset was experimentally validated, and the effect of high-frequency features on the performance of NILM was systematically evaluated. These contributions provide significant advancements in the monitoring of energy and enable the development of sophisticated tools for smart energy management.

This paper is organized as follows: Section 2 provides a review of related work and NILM datasets available in 2025. Section 3 details the design of our measurement system. Section 4 describes the data acquisition methodology and processing pipeline and presents and characterizes the resulting dataset. Section 5 evaluates NILM algorithms using our dataset. Section 6 discusses the results, limitations, and future directions. Section 7 concludes with a summary of our main contributions.

## 2. Related Work

### 2.1. Existing NILM Datasets

NILM research relies mainly on the availability of high-quality datasets for algorithm development. Some datasets that have been introduced include the Reference Energy Disaggregation Dataset (REDD [6], released in 2011), which has been essential in establishing NILM research and providing both whole-house and circuit-level power measurements for multiple homes. Moreover, it has limited high-frequency captures and often features discontinuous monitoring over time. Subsequent datasets like the UK Domestic Appliance Level Electricity (UK DALE [7], released in 2014) was a major step forward, with long periods of measurement across multiple homes and at a higher temporal resolution. But, the granularity of data at the individual appliance level remains limited, which constrains certain types of fine-grained NILM analysis. Meanwhile, the Building Level fUlly labeled dataset for Electricity Disaggregation dataset (BLUED [8], released in 2012) took a different approach, focusing on detecting and labeling individual appliance switching events rather than capturing continuous operation. While these datasets brought valuable advancements, most had to compromise between either short bursts of high-resolution data or extended low-frequency monitoring, leaving a persistent gap in continuously available high-frequency recordings. More recent datasets have struggled to fill some of the gaps left by previous studies. The Almanac of Minutely Power dataset (AMPds on 2013) [9], for example, pursued long-term monitoring, with one-minute resolution and relatively low power resolution, but allowed the monitoring of continuous loads over a longer time frame. A comparative overview of these datasets is presented in Table 1.

### 2.2. High-Frequency Data and Harmonic Analysis in NILM

An emerging area of NILM research includes the use of high-frequency electrical characteristics to identify appliances. The use of harmonics in particular has proven to be effective in distinguishing between appliances that have similar power usage but different internal circuits. Studies, such as those of [10,11], have shown that analyzing harmonic content during both steady-state operation and transient events gives highly discriminative features, particularly for modern electronic devices with nonlinear power characteristics. Transient events (appliance start-up/shut-down) captured at high sampling rates also provide distinctive signatures [12]. To extract these harmonic features, Fast Fourier Transform (FFT) analysis has become a standard method. Researchers have examined various preprocessing techniques and feature selection methods to improve classification performance. For example, refs. [13,14,15] showed that combining time domain and frequency domain features together helps in producing better results than using either one alone, as devices have similar power modes. Recently, models like the Neural Fourier Energy Disaggregator (NFED) [16] have used Fourier transforms within deep learning architectures to improve NILM efficiency. Alongside this, Athanasiadis et al. [17] proposed a real-time, event-based NILM method using CNNs, offering a lightweight alternative for real-time disaggregation. An example of a promising method is described in [18], where V-I trajectory mapping was performed to capture the relationship between instantaneous voltage and current. This method is primarily used for identifying devices with reactive components or with a specific transient behavior, such as during device starting. The separate techniques mentioned above require high-resolution sampling, which exceeds the capabilities of most existing public datasets.

### 2.3. Gaps in Current Public Datasets

Despite advances in NILM research, there are still many gaps in available public NILM datasets. There is especially a shortage of datasets that offer continuous high-frequency sampling, detailed harmonic analysis, and long-term monitoring across a diverse range of households. High-precision measurement instruments are costly and difficult to install, which is why many high-resolution studies have been conducted in laboratories with small-scale experiments. Additionally, the enormous volume of high-frequency data makes long-term storage and processing more difficult, resulting in using, in some cases, selective sampling techniques, which potentially cause appliance key behaviors to be missed. This has hampered the development of more advanced NILM algorithms that could rely on richer electrical signatures. In response to these limitations, we present a complete and reproducible data acquisition system. To encourage contributions and the future expansion of such datasets, all associated hardware and software resources are publicly available on the shared repository.

## 3. Design of the High-Frequency Measurement System Using the ATM90E36A

### 3.1. System Architecture Overview

**Figure 1:**
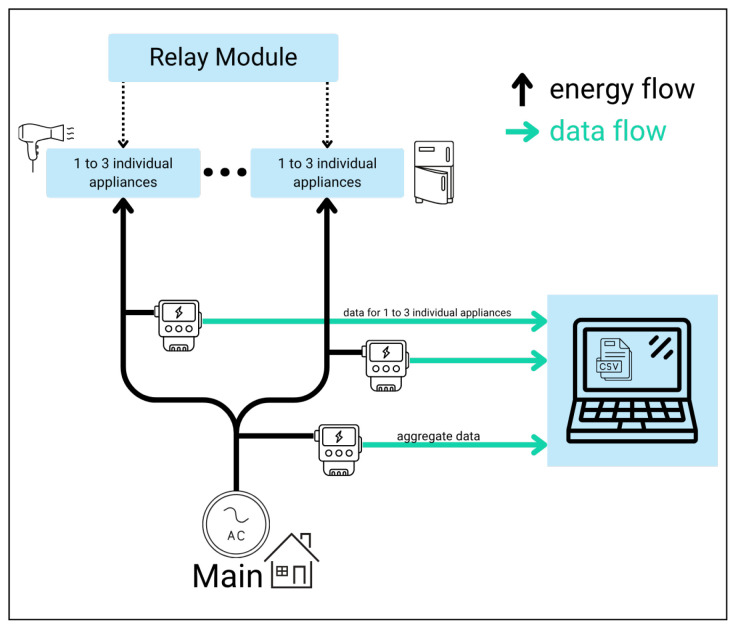
Block diagram of the experimental setup.

Capturing detailed electrical signatures that can advance NILM applications is a major challenge. It needs a robust and reliable data acquisition system, which requires careful consideration of how to select the components, signal integrity, and systems architecture. In this study, our hardware implementation, as illustrated in the block diagram in Figure 1, used a commercially available board [19] based on the ATM90E36A energy metering integrated circuit (IC) (DitroniX Ltd., Maidstone, UK) [20]. This choice allowed for replicability and broader adoption. Besides using these components, we are developing our own custom sensing cards. These cards are currently in the prototype stage. In the following, we will detail the overall system design, key components, and integration considerations that enable the capture of high-resolution electrical parameters for NILM research.

**Figure 2:**
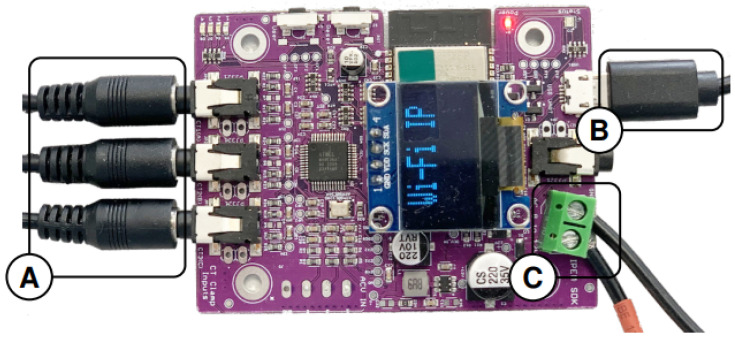
The data acquisition node, showing (A) inputs for CTs, (B) USB port for serial communication, and (C) 12V AC input for power and voltage reference.

Our measurement setup consists of four identical sensing nodes. The number of sensor nodes is completely scalable depending on the number of devices to record. For instance, as shown in Figure 2, each individual sensing node has three inputs for current transformers (CTs), meaning that a single node can simultaneously monitor up to three different appliances. Therefore, increasing the number of installed nodes directly increases the number of monitored points. Each is capable of measuring both aggregate household consumption or individual power appliance(s). Each sensing node consists of one ATM90E36A based an energy metering board connected to an ESP32 microcontroller, which is responsible for performing data acquisition, processing, and transmission to a storage device.

### 3.2. Core Metering IC: ATM90E36A

The measurement system’s core is the ATM90E36A integrated circuit [20], which is the central component of the data acquisition node shown in Figure 2. This IC provides multiple benefits compared to alternative metering technologies, including true RMS voltage and current measurements for three phases, active and apparent power, power factor, and line frequency calculations, which makes it well-suited for sophisticated energy monitoring. One of the basic characteristics of the ATM90E36A is that it has an onboard hardware Discrete Fourier Transform (DFT) engine that generates up to 32nd-order harmonic analysis. We showed in [15] that it is sufficient to compute odd harmonics up to the 15th order in order to discriminate most devices regarding their harmonic behavior. Using higher-order harmonics does not improve the classification, whatever the classifier. The ATM90E36A, being capable of sampling at $F_{e}=8$ kHz and calculating up to the 32nd-order harmonic component, was a perfect choice for our project. Using a window of 0.5 s was sufficient to collect a data sample of size N=4000, ensuring a resolution equal to $\frac{F_{e}}{N}=2\;\mathrm{Hz}$. The duration of the window was a compromise to obtain good resolution but also a sampling rate for the computation of harmonics high enough (about 2 s, 1.5 s being the time to format and send data to the computer or through the network). In contrast with other systems where time-consuming FFT calculations are carried out by an external microcontroller, the onboard hardware DFT engine in this case extracts frequency domain features very efficiently, thereby facilitating accurate appliance identification based on harmonic signatures. Although the ATM90E36A is a cost-effective solution, it presents certain limitations. Its 8 kHz sampling rate is sufficient for many NILM applications focused on steady-state harmonics, but it may not capture the fastest transient events. Moreover, while the integrated DFT engine provides the magnitudes of the harmonic components, it does not offer their phase angles.

Class 0.2S meters offer high accuracy, with a percentage of error as low as ±0.2%, making them suitable for the measurement of energy. Additionally, they have also built-in calibration features that allow for improved accuracy and sensor stability over time. Consequently, these capabilities allow for instantaneous high-frequency monitoring without having too heavy a workload on the external processor. Therefore, these sensors are useful not only for utility scale systems that need accuracy but also for residential purposes, especially in NILM systems aimed at tracking household energy use more effectively.

### 3.3. Microcontroller: ESP32 and Data Handling

**Figure 3:**
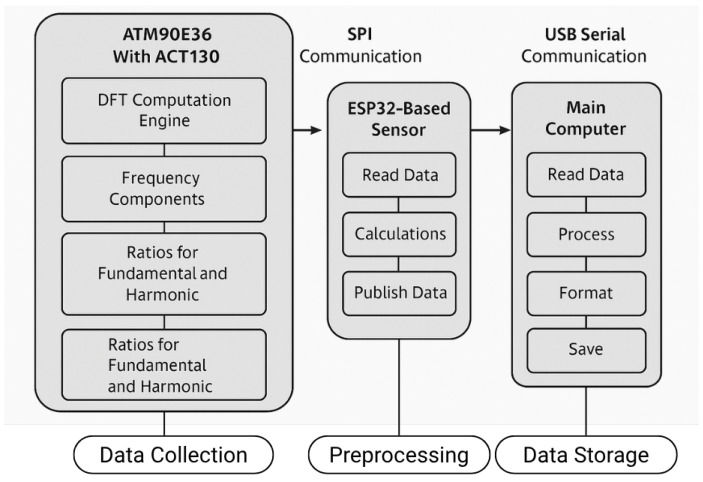
Measurement system diagram.

The well-known ESP32 microcontroller (DitroniX Ltd., Maidstone, UK) is used to control the ATM90E36A and perform data transmission through the network or using the serial port for data acquisition, preprocessing, and communication for the system, as illustrated in the overall system architecture in Figure 3. By exploiting its dual core architecture and extensive peripherals set to manage multiple tasks, it establishes high-speed SPI communication with ATM90E36A energy metering IC and uses Direct Memory Access (DMA) for streaming seven-channel ADC raw data with minimal CPU overhead to achieve a maximum sampling rate while maintaining system responsiveness. In every sensing node, ESP32 performs real-time calculations and arranges and formats the processed data for serial transmission to a central logging laptop. The processed data is then saved in compatible file formats for later analysis. This structured data pipeline improves data reliability and integrity, in addition to minimizing the risks linked with potential losses that may occur due to delays in processing or communication instability. Further, the microcontroller’s onboard Wi-Fi module offers data transmission and remote configuration in real time, thereby making both remote and live monitoring possible. In cases where network connectivity is poor, an onboard microSD card offers local storage that can sustain over 23 weeks of uninterrupted measurements on a standard 1 GB microSD card.

### 3.4. Sensors and Current Measurements

The measurements of the electrical current in the system were taken using split-core current transformers (CTs). Those CTs were selected to handle the expected loads at each monitoring location. In our case, CTs were chosen with a rated input current of 100 A and rated output current of 50 mA for both aggregate and measured individual appliance loads. They were appropriate to measure the anticipated load range while avoiding loss in resolution. Any CTs from diverse manufacturers, with diverse rated input currents, required appropriate calibration so that the accuracy of measurements and consistency across the dataset were ensured. Specifically, the system was based on the current transformers (DitroniX Ltd., Maidstone, UK), which work by sensing the alternating current (AC) magnetic field when passing a conductor through the CT. The CTs can measure up to 100 A and provide an analog output directly proportional to the primary current so that electrical consumption can be recorded. The design of the CTs is a clamp-on style to prevent risks that come with direct contact with the conductor. The clamp-on design also enables the user to install it safely and non-intrusively.

### 3.5. Power Supply

To ensure phase correctness between voltage and current measurements (which is very important for power factor and harmonic analysis), the voltage reference was directly connected to the ATM90E36A. This avoided the phase shifts that were possible with using a voltage transformer. The power supply of the subsystem was designed to maintain measurement accuracy. Each node used an isolated AC/DC converter to avoid ground loops and common-mode interference, thus maintaining signal quality. The measurement system was powered by a Greenbrook DAT01A AC Bell Transformer (Greenbrook Electrical, Harlow, UK) (15 V at 1 A), which served both as the primary power source and the voltage reference for measurements. It accepted an AC 220 250 V input and gave output at 4 V, 8 V, and 12 V. This system was operated at 12 V for safety and reliability purposes. Any such transformer may be used. It still enabled the system to be stable and avoided potential electrical risks. By making these design and implementation decisions, we ensured that we were capable of collecting accurate measurements and preserving the electrical characteristics of load consumption for further analysis.

### 3.6. Relay Module for Controlled Appliance Switching

The relay module is a crucial component for mimicking the real-world use of an appliance as it allows for the controlled switching on and off of the connected appliance. The relay can utilize both randomized switching patterns and user-generated custom schedules to create a wide variety of different conditions. The more varied the different situations, the wider the range of load signatures that can be captured, and NILM models developed and tested based on overlapping appliance usage and dynamic ON or OFF patterns over time will provide representations that resemble everyday household patterns. By automating appliance control, the relay module allowed for consistency, reproducibility, and control, which was essential for all the data collected for subsequent data comparisons and model validation. Furthermore, this method allows for the more thorough testing of NILM algorithms in more complex and realistic situations, which can improve the robustness and generalization of the models.

The design choices detailed above resulted in a system that is both powerful and cost-effective. This was realized by using the affordable ATM90E36A IC, which has an onboard DFT engine to process a high-frequency 8 kHz signal to extract the harmonic signatures. So, instead of storing the voluminous raw waveform, which can lead to enormous data generation, we only captured this compact and processed harmonic data at a much lower frequency. This entire process was managed by a low-cost ESP32 microcontroller, avoiding the need for costly external processors.

## 4. Data Acquisition and Processing

### 4.1. System Calibration

There are numerous challenges to acquiring and processing high-frequency electrical data that involve hardware, communication protocols, software architecture, and storage management. In this section, we will present an overview of the methodology for the acquisition, synchronization, processing, and storing of the electricity measurements captured thanks to the ATM90E36A system. The data acquisition process began with adjusting the internal registers of the ATM90E36A to obtain the desired measurement parameter and sampling characteristics. Each measurement obtained from the IC was composed of many metrics, including voltage, current, active/reactive power, apparent power, frequency, power factor, and phase angle, as detailed in the device specifications presented in Table 2.

With advanced harmonic analysis capabilities, the system included Total Harmonic Distortion (THD) metrics and harmonic components up to the 32nd order with an embedded DFT engine operating with an 8 kHz input sampling rate. During the process of data collection, readings were adjusted as needed based upon reference equipment; in our case, we used the Fluke 1738 Power Logger to ensure consistent measurement with load variations.

### 4.2. Data Handling and Transmission

To build an accurate dataset for disaggregation analysis, we had to ensure that the timestamp values between all the measurement nodes were aligned. The proposed system has a high-resolution timer, which gave us the ability to timestamp events when the sensors acquired new data. We used a Python 3.9 script (available at https://github.com/fariddinar/nilm-dataset (accessed on 20 June 2025) based on its multiprocessing capabilities to manage data streams from the four distinct serial ports simultaneously. Each port was dedicated to a specific set of electrical measurement phases, in our case, COM9 for data in phases 1, 2, and 3; COM11 for phases 4, 5, and 6; COM10 for phases 7, 8, and 9; and COM8 for phases 10, 11, and 12. For each COM port, an independent subprocess was assigned. This subprocess continuously monitored its port and read incoming data packets from the sensor node. This method prevented the delays issued in sequential processing, ensuring efficient, real-time data capture from all four sensors. We also used the same Python environment to process the captured data. We began by organizing the collected readings based on their phases, since each serial port provided us with data from three different phases. Then, measurements from different nodes were aligned, and the resulting data was exported in a structured format, with each output file corresponding to a specific phase. The detailed file contents is described in the following section. We also provided metadata that included more context of the dataset, such as calibration parameters and additional information about the appliances. By exploiting modern embedded systems capabilities as well as a software architecture, this approach provided reliable measurements across multiple monitoring points. A key methodological consideration was how to handle the timestamps from these independent data streams. At the beginning of our project, we sought to synchronize the different measurements from the different acquisition cards. Each card transmitted, every two seconds, a measurement of the voltage, current, power, and harmonics on its three channels. These measurements were synchronous with each other and time-stamped. In order to obtain a uniform time-stamp, we chose a card that would provide the time reference and resampled the other cards by interpolation (linear or nearest neighbor). This preliminary step was intended to help the different models in their classification task. However, this step introduced errors due to interpolation. By comparing the results with the interpolated values and the results keeping a separate time-stamp, we did not notice any difference in the quality of the predictions. We therefore stopped recalibrating the data in time and kept a separate time-stamp in the files, which represented non-interpolated (and therefore unbiased) values. Nevertheless, the code for the time shifting method we evaluated is included in the provided resources.

### 4.3. Dataset Characteristics and Analysis

#### Dataset Structure and Contents

The dataset created in this work represents an advancement in the feature richness available for energy disaggregation studies. While this initial release does not constitute long-term monitoring, its primary purpose is to present a system capable of acquiring such data continuously over extended periods. The dataset contains a limited number of appliances; they were selected to cover a variety of load types, including resistive (iron, lamp), motor-driven (hair dryer), and electronic (laptop charger, screen) loads.

The dataset was structured to optimize both storage efficiency and analytical accessibility. At a higher level, measurements were organized by four distincts nodes (sensors), each corresponding to different physical monitoring points within the system. Each node recorded multiple phases (channels), assigned either to an individual appliance or to an aggregate circuit grouping. The recorded circuits were as follows (in this notation, S1 refers to Sensor 1 and P1 refers to Phase 1): Node S1 monitored a hair dryer (P1), an electric water heater (P2), and an aggregate circuit (P3). Node S2 included a hair straightener (P4) and a fridge (P5). Node S3 recorded measurements from an iron (P7) and a screen (P8). Finally, Node S4 collected data from a laptop charger (P10) and a lamp (P11).

We generated a single CSV file for each measurement phase, naming it according to its corresponding node and phase, for example, S1P1.csv, S1P2.csv, etc. All CSV files contained the following columns: time (timestamp of the measurement), irms (RMS current, I_RMS_), vrms (RMS voltage, V_RMS_), power_factor (power factor, cosφ), p_apparente (apparent power, P_apparente_), p_active (active power, P_active_), and h1 to h32 (the 1st to 32nd current harmonic amplitudes, also denoted h_1_ to h_32_, see (3)), which were a direct output of the ATM90E36A’s onboard DFT engine. The theoretical basis for these harmonic components relied on the Discrete Fourier Transform (DFT). Let x(t) be a signal and let $F_{e}=\frac{1}{T_{e}}$ be the sampling frequency and N the size of a sample. The Discrete Fourier Transform (DFT) coefficient of order k is given by

(1) $${\hat{x}}_{k}=\sum_{n=0}^{N-1}x\left(nT_{e}\right)e^{-2i\pi \frac{kn}{N}}$$

If x(t) is a T-periodic signal with frequency F being a multiple of $\frac{F_{e}}{N}$, i.e., $F=\nu \frac{F_{e}}{N}$, then

(2) $${\hat{x}}_{k}=\left\{\begin{array}{cc} \frac{N}{T}\int_{0}^{T}x\left(t\right)e^{-2i\pi \frac{jt}{T}}\;dt & \mathrm{if}\;k=j\nu ,\;\mathrm{for}\;\mathrm{an}\;\mathrm{integer}\;j\\ 0 & \mathrm{if}\;k\;\mathrm{is}\;\mathrm{not}\;a\;\mathrm{multiple}\;\mathrm{of}\;\nu \end{array}\right.$$

Hence,

(3) $$h_{j}=\frac{1}{N}\left|{\hat{x}}_{j\nu }\right|$$

This theoretical framework supports the extraction of harmonic information. In our specific data acquisition setting, the ATM90E36A IC operated with a sampling frequency Fe=8kHz and the DFT engine processed a sample window of N=4000 samples (the DFT period was 0.5 s) to compute these harmonic components. The ATM90E36A directly provided the magnitudes of the harmonic coefficients $h_{1}$ to $h_{32}$.

### 4.4. Dataset Analysis and NILM Potential

**Figure 4:**
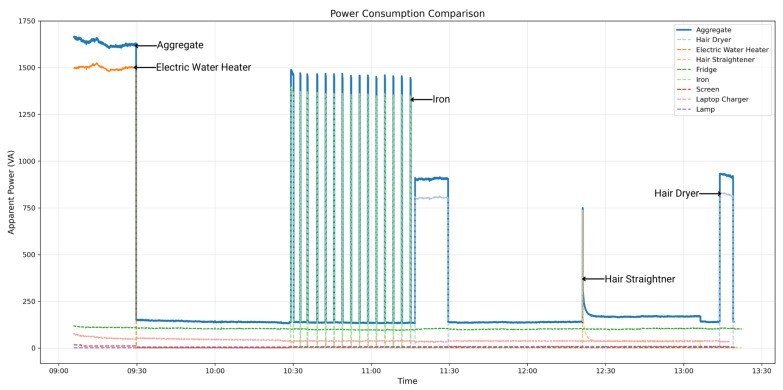
Apparent power consumption for aggregate and individual appliances.

The data we gathered showed how we could visually differentiate the energy consumption of every appliance. It gave us an idea of how every device was behaving when in use, making it suitable for NILM applications. In Figure 4, apparent power consumption changes show distinctive usage patterns for the appliances. For example, the iron showed the effect of its thermostatic cycling, with periodic spikes when heated, followed by periods of inactivity. The hair straightener and hair dryer, in comparison, had short impulses, indicating that they were manually switched on and off. In comparison, the fridge (it was a small fridge without freezer, so it had continuous power consumption, unlike a classical fridge which presents some periodic activation cycles) and the lamp showed more continuous and stable consumption profiles. The total power signal also had significant transitions, especially when high power devices like the electric water heater turned on.

In addition to power levels, the dataset had a high number of features. Even though they are not not explicitly shown in this plot, devices such as a screen or laptop charger were frequently invisible in aggregate traces due to their low power consumption. However, their electronic components produced a known harmonic content that was (for some devices) characteristic. The frequency domain features provided extra appliance-specific information that was not obtainable through mere observation of power use. These patterns confirmed the capability of the dataset in identifying the actual behavioral characteristics of the majority of home appliances, thus offering useful training and validation data for NILM algorithms.

A harmonic analysis of different appliance categories provides meaningful features for disaggregation. Resistive loads like incandescent lights and heating devices create very low harmonic distortion, with power concentrated mainly in the fundamental component. Motor-driven appliances generate distinctive harmonic profiles corresponding to the operating state, and electronic appliances with switch-mode power supplies generate richer harmonic signatures up to a high order. Spectrogram heatmaps illustrate how different appliance classes occupy characteristic regions in the harmonic space, suggesting valuable feature spaces for classification algorithms.

**Figure 5:**
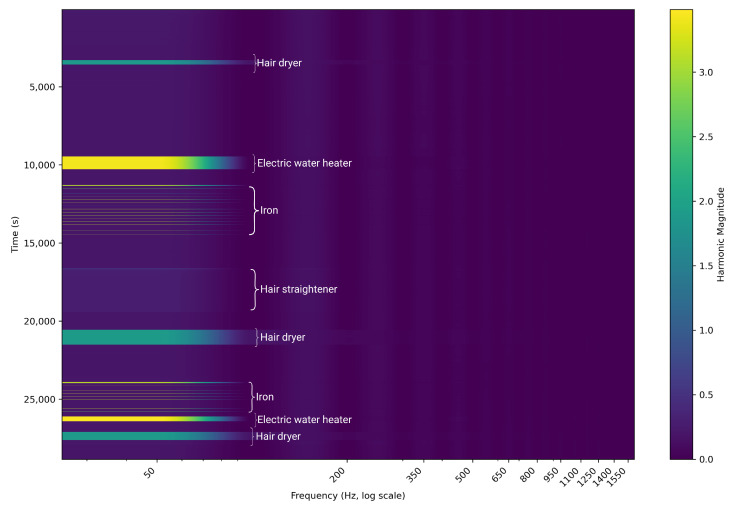
Harmonic content over time for individual appliances. The y-axis is time, the x-axis is frequency (log scale), and color intensity represents harmonic magnitude.

**Figure 6:**
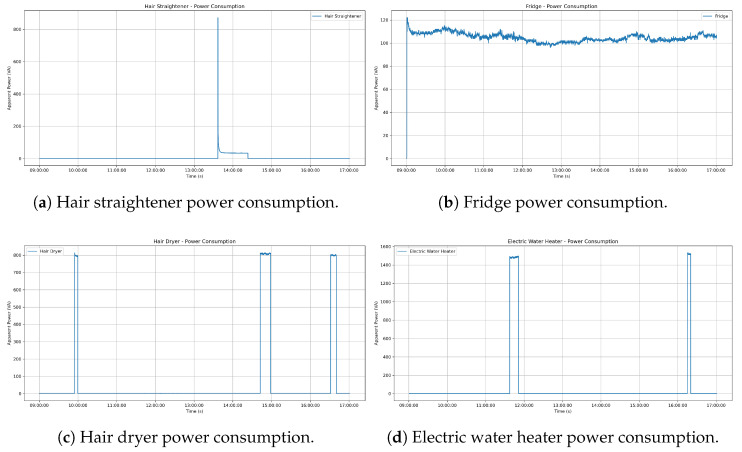
Apparent power consumption over time for some individual appliances.

The rich content provided by this dataset was also illustrated by visualizing the temporal behavior of the harmonic spectrum, shown in Figure 5. It is a spectrogram representation with time represented on the y-axis, frequency (harmonic order) on the x-axis, and color intensity corresponding to harmonic magnitude and identifying operational signatures patterns. For example, motor-driven appliances like the ’hair dryer’ showed characteristic changes during their startup, likely capturing the mechanical loading. In the case of resistive loads with thermostatic control, like the ’iron’, we could observe periodic impulses of harmonic energy concentrated primarily at the fundamental frequency (50 hz) and maintaining a spectral signature during every heating cycle. Electronic devices like the hair straightener could have a much more complex and likely much wider distribution of harmonics that changed while operational. Similarly, we could differentiate appliances that had the same steady-state power over time by observing these dynamic harmonic signatures. Distinct appliance-level usage patterns are also illustrated in Figure 6.

## 5. Performance Evaluation and NILM Model Testing

To validate the utility of our newly developed dataset for a NILM application, we conducted a comprehensive experiment on the performance of machine learning algorithms in disaggregation. This section builds upon our recent work [3], extending the analysis by applying the established methodology to a much larger and more diverse dataset (which was not available at that time). Our primary objectives were to evaluate model performance with this new data, compare results for appliances previously studied (hair dryer and iron), and evaluate performance on newly included appliances (electric water heater and hair straightener).

### 5.1. Experimental Setup and Dataset Configuration

We focused on four domestic appliances selected for the diversity of their operational parameters and energy consumption profiles: the hair dryer, iron, electric water heater, and hair straightener. The hair dryer and iron were chosen to allow for direct comparison with our previous results, while the electric water heater and hair straightener represented an expansion of the evaluated appliance set, introducing different load characteristics. The NILM models were trained using a large volume of data from the dataset detailed in Section 4 of this paper, with resources available at https://github.com/fariddinar/nilm-dataset (accessed on 20 June 2025). Specifically, the training set comprised multiple recording sessions collected on different days (“05–12 8 h”, “05–14 8 h”, “05–15 8 h”, “05–16 8 h”, and “05–22 8 h”), totaling approximately 40 h of training data. To ensure a robust assessment of generalization, model performance was tested on an entirely separate 8 h recording session (“05–19 8 h”), which was not seen by the models during training. This extensive training and testing regime on our new dataset provides a more challenging and realistic benchmark compared to the small dataset used in our prior work.

### 5.2. NILM Models, Features, and Learning Strategy

To maintain consistency, and using the new dataset, the learning framework, features, and model architecture were identical to those described in our previous work [3]. We adopted the sequence-to-point (Seq2Point) learning framework. This method uses a sliding window of aggregated electrical measurements to predict the power usage of a target device at the midpoint of the window. The following regression models were benchmarked: Random Forest (RF), which is an ensemble learning method known for its robustness and effectiveness with time series feature sets; a Convolutional Neural Network (CNN), comprising five convolutional layers designed to capture temporal patterns; and a Support Vector Machine (SVM), implemented as Support Vector Regression (SVR) for this regression task.

### 5.3. Evaluation Metrics

The disaggregation performance of each model was quantified using three standard NILM metrics: Mean Absolute Error (MAE) (4), Mean Relative Error (MRE) (5), and Mean Squared Error (MSE) (6), identical to those in our previous evaluation, to facilitate direct comparison.

(4) $$\begin{array}{c} \mathrm{MAE}=\frac{1}{N}\sum_{i=1}^{N}\left|p_{i}-{\hat{p}}_{i}\right| \end{array}$$

(5) $$\begin{array}{c} \mathrm{MRE}=\frac{1}{N}\sum_{i=1}^{N}\frac{|p_{i}-{\hat{p}}_{i}|}{|p_{i}|} \end{array}$$

(6) $$\begin{array}{c} \mathrm{MSE}=\frac{1}{N}\sum_{i=1}^{N}{\left(p_{i}-{\hat{p}}_{i}\right)}^{2} \end{array}$$

In these metrics, $p_{i}$ represents the ground truth power of an appliance at time step *i* and ${\hat{p}}_{i}$ is the corresponding predicted power from the model.

### 5.4. Results and Discussion

**Figure 7:**
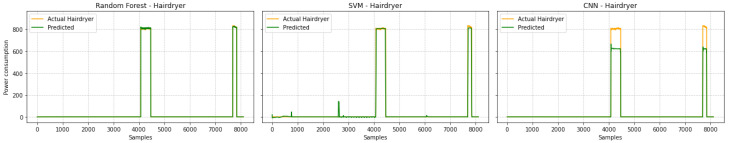
Hair dryer usage prediction by different models on the new dataset. The orange line is the actual power and the green line is the predicted power.

**Figure 8:**
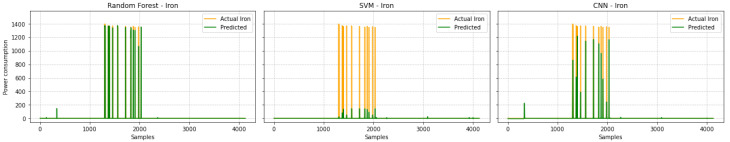
Iron usage prediction by different models on the new dataset.

**Figure 9:**
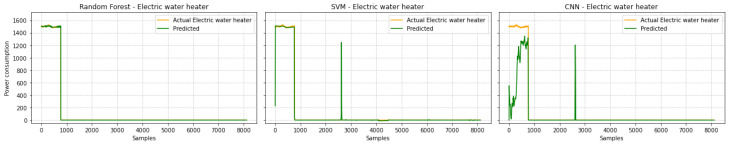
Electric water heater usage prediction by different models on the new dataset.

**Figure 10:**
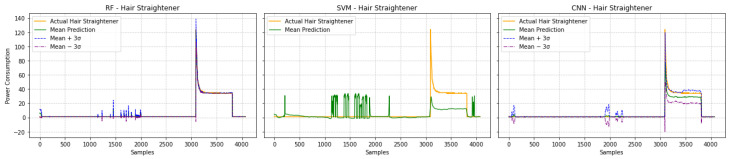
Hair straightener usage prediction by different models on the new dataset.

This section presents the quantitative performance of the RF, SVM, and CNN models for disaggregating four target appliances from the aggregate signal (illustrated in Figure 4). A summary of the performance metrics on the test dataset is presented in Table 3. Visual comparisons of the predicted power consumption against the ground truth for each appliance further illustrate the models behaviors, as shown by the hair dryer predictions in Figure 7, iron predictions in Figure 8, electric water heater predictions in Figure 9, and hair straightener predictions in Figure 10. In the last figure, we included a confidence interval of ±3σ, where σ is the standard deviation computed across 10 independent runs.

#### 5.4.1. Comparison with Previous Work (Hair Dryer and Iron)

We first examined the hair dryer, an appliance also included in our prior study. For the hair dryer, RF’s performance was notably good (MAE: 0.42, MRE: 0.033). This accuracy is visually confirmed in Figure 7, where the RF predictions (in green) almost perfectly overlay the actual power consumption (in orange) during the appliance’s operational activation. In contrast, the CNN model (MAE: 13.16), as seen in its respective subplot in Figure 7, while correctly identifying the hair dryer’s operational periods, consistently underpredicted the peak power. The SVM (MAE: 4.63) also showed significant performance for predicting the actual consumption.

The iron, another appliance from our previous study, showed periodic heating cycles. For the iron, RF achieved an MAE of 1.88. Figure 8 illustrates RF’s performance in accurately tracking the characteristic periodic heating cycles of the iron, capturing both the timing and the power magnitude of these power activations. In contrast, the SVM did not achieve comparable precision for this dataset. The CNN model also demonstrated a strong performance in detecting the start and the end of the iron’s periodic heating cycles but struggled with accurately estimating its power consumption.

#### 5.4.2. Performance on Newly Added Appliances (Electric Water Heater and Hair Straightener)

The evaluation was extended to two new appliances not included in our prior work: the electric water heater and the hair straightener. The RF model’s strong performance extended to the newly evaluated appliances. For the electric water heater, RF achieved a remarkable MAE of 0.46 and an MRE of 0.037. Figure 9 demonstrates RF’s ability to accurately disaggregate this high-power appliance during the heating cycle. The SVM also accurately followed the actual power usage but generated false predictions when the appliance was inactive. In contrast, the CNN struggled with this appliance (MAE: 69.05). Its predictions showed considerably higher errors while attempting to predict the actual usage.

For the hair straightener, another device with distinct on/off usage but lower power, RF again led with an MAE of 0.27 and an MRE of 0.113. Figure 10 shows the RF predictions, which accurately captured power consumption when the appliance was in use. The CNN, with an MAE of 1.73, also identified the event timings, with less precise regression of the specific power level. In contrast, the SVM showed higher errors (MAE: 7.77). Its predictions were marked by some noise during off-periods and a less accurate estimation of the power when the appliance was active.

### 5.5. Discussion of Overall Model Performance

The consistent and dominant performance of Random Forest, which achieved the best overall results across all four appliances on this large dataset, is a key finding. In our earlier experiments with a limited dataset [3], Random Forest (RF) was a competitive method that often outperformed others. The current results strongly suggest that the combination of the rich harmonic and electrical features captured by our ATM90E36A-based system, coupled with RF’s ensemble nature, facilitates effective learning and robust generalization. This aligns with the existing literature [21], which reports RF outperforming other supervised regression methods for NILM by averaging multiple decision tree outputs, and so mitigating overfitting characteristics, which is particularly beneficial when dealing with carefully selected, high-dimensional feature subsets such as those used here. RF also required fewer hyperparameter tunings and had faster training times compared to the CNN and SVM models, which further illustrates its effectiveness under circumstances with a small dataset [21]; our previous results confirm this effectiveness, which is even enhanced when applied to a larger dataset. Despite having access to a large dataset, which typically benefits deep learning models, the CNN did not outperform RF in this regression task. This could have been due to several reasons, such as the specific CNN architecture used, the fact that the feature space was already highly informative to RF, or the need to tune the CNN’s hyperparameters. The SVM produced the highest number of errors in terms of both MAE and MSE, confirming its limitations for precise power regression with this type of multi-feature data.

In summary, training on our multi-day dataset showed the Random Forest model’s ability to achieve accurate disaggregation results for all tested appliances. This highlights the value of large, diverse datasets in NILM research and makes RF, with appropriate feature engineering, a powerful benchmark for this task. Recognizing the potential of deep learning, future work will investigate more advanced and potentially more efficient CNN architectures, along with rigorous hyperparameter optimization strategies, to explore if their performance can be elevated to surpass or complement the strong baseline established by Random Forest on this rich dataset.

## 6. Discussion and Future Work

The development and analysis of this NILM dataset reveals both promising opportunities and persistent challenges for energy disaggregation research. This section provides context for the results within the NILM ecosystem and suggests opportunities for future work. The impact of high-frequency monitoring on NILM performance suggests that electrical signatures contain more value than power metrics alone. Active and apparent power approaches struggle to differentiate appliances within households with similar consumption patterns, and they have even more difficulty when multiple appliances are switched on simultaneously. Including harmonic content allows us to overcome the limitation of monitoring only active and apparent power because any given appliance has a distinct electrical signature resulting from differences in internal circuitry. This approach proves especially valuable for modern households where an increasing proportion of energy consumption comes from electronic devices with similar power levels but distinctive harmonic signatures. The performance improvements observed across different classification algorithms confirm that these high-frequency features provide generalizable benefits rather than algorithm-specific advantages. Our results suggest that future NILM methods could combine richer feature sets even when working with basic classification approaches, as the distinctive information contained in harmonic components enhances disaggregation performance. However, it is important to note that the collected data, according to our approach, did not allow us to characterize transient phases at a time scale of less than one second, which are characteristic of the start-up and, to a lesser extent, shutdown of certain devices. Indeed, on the one hand, we chose to alternate data logging and data processing (even though the ATMEL processor operated in DMA), which simplified real-time constraints. On the other hand, we used a Fourier approach that assumed stationarity for short periods (0.5 s), whereas an approach focused on transients would have required the use of tools that took non-stationarity into account (wavelet transforms, timing diagrams). Nevertheless, we consider that methods based on startup and shutdown detection may lack robustness, as a device startup can always be missed. Moreover, a database allowing for this detection would require regular sampling at a minimum of 2 kHz (allowing spectrograms to be plotted showing harmonics up to the 15th order) continuously, which represents for each channel a volume of 691.2 MB in single precision per day, compared to 6.26 MB for our approach (38 float values every two seconds). Thus, our methodology is not compatible with transient detection when transient phenomena occur at the millisecond scale. Transient analysis emerges as a particularly valuable approach for appliance identification, often providing more distinctive signatures than steady-state operation. These transient events happen in too short a time to be detected with traditional low-frequency monitoring, but are easily recognizable in high-resolution data. Future NILM can adopt hybrid approaches that use transient detection for identification and steady-state monitoring for tracking an appliance’s operational state. Despite these advantages, there exist a couple of technical limitations worth highlighting and provide opportunities for improvement in future work. The current system needs accurate initial calibration at each measurement node for consistent results across sensors and current transformers. Furthermore, we did not plan to detect or compensate for potential phase misalignment or voltage variations that could occur during long-term data collection. Taking these fluctuations into account remains the responsibility of the classifiers, which have all the necessary measurements to handle them, in particular active and reactive powers and cosφ. An advanced automated calibration technique would enable wider usage in varied environments without compromising measurement accuracy.

The demands of computational power and data storage impose significant practical limitations on high-frequency monitoring. The proposed framework generates about 130 MB of data in a day across 12 channels, demonstrating storage efficiency and research adequacy. If used in actual home environments, such as across more than 10 houses, each with an average of 30 appliances, this would lead to enormous data generation. For example, if 1 household generated around 300 MB daily of data, 10 households would generate close to 3 GB daily, demonstrating the scalability and feasibility of the system for developing a large-scale monitoring dataset.

New processing methods present a promising solution to this challenge by performing feature extraction and event detection locally on the measurement nodes, sending only data that is useful rather than raw measurements. The initial experiments with the application of a simple harmonic analysis algorithm executed directly on the IC demonstrated the feasibility of extracting frequency domain features with relatively minimal additional hardware. Future works will look into more advanced processing techniques, either through specialized signal-processing ICs or more powerful microcontrollers to enable local processing of preliminary classification stages to enable the creation of real-time NILM. Although the dataset demonstrates strong potential for NILM applications, it was collected using a specific set of appliances. No bias was observed within this scope. However, generalization to other contexts, particularly those with different voltage standards or higher current loads, should be approached with caution. For instance, our setup used 100 A:50 mA CT, which would need to be adapted in environments where current levels exceed 100 A. While the current dataset was focused only on residential buildings, commercial and industrial environments also present interesting opportunities for energy disaggregation, with potentially adapted approaches due to their different appliance types, load characteristics, and usage patterns. Expanding high-frequency monitoring to include non residential buildings would allow researchers to explore how harmonic based methods perform across a broader range of real-world scenarios and help devise NILM approaches to diverse operational environments. Integration with other sensing modalities represents a particularly promising direction for future work. While electrical measurements provide rich information about appliance operation, complementary data from environmental sensors (temperature, humidity, occupancy), smart meters (gas, water consumption), or even acoustic monitoring could further enhance disaggregation accuracy. Other datasets that include multiple types of data could help develop smarter disaggregation methods by linking electricity use with other factors in the building [22].

Within the context of smart energy management, this work clarifies how advanced load disaggregation makes possible advanced applications beyond energy monitoring. By breaking down the consumption of individual appliances, users can gain knowledge about energy intensive appliances and change-related use behaviors accordingly, resulting in energy savings. Another aspect is the ability to identify different modes of operation for devices, which creates a foundation for predictive warnings due to the detection of abnormalities or failures. This aspect further opens doors to personalized energy efficiency advice based upon empirical use behaviors. At the system level, this would help enable automated demand response systems requiring the smart adjustment of loads according to functional importance in preference to traditional simple power thresholds. All of these added functionalities are made possible by enhanced accuracy through the disaggregation that high-frequency monitoring enables, emphasizing how fundamental measurement capabilities maximize end-user benefits within intelligent energy systems.

## 7. Conclusions

In this study, we presented the development, implementation, and analysis of a dataset created using a metering system based on the ATM90E36A IC. In each step from hardware setup and synchronization to data collection and analyzing algorithms, every part was necessary to examine how electrical features, specifically harmonics, can improve energy disaggregation and appliance tracking. The resulting dataset provides a valuable resource for advancing NILM by offering a detailed set of features across both time and frequency domains. The hardware implementation overcomes technical challenges associated with data acquisition, while maintaining a solution that is cost-effective and suitable for widespread deployment. The distributed nature of the system with synchronized measurement points allows for both the aggregate measurement as well as the individual measurements to provide ground truth in identifying and validating the algorithms. Analysis of the collected data shows the advantage of using harmonic content to identify appliance consumption. The system’s ability to extract harmonic parameters up to the 32nd order allows for the identification of unique signatures that remain stable. This is useful when multiple appliances are operating at the same time, addressing one of the main challenges in NILM research. The results also show how the measurements taken at a higher frequency capture some information that is often not available by traditional, lower-resolution datasets. This additional information helps to explain the improvements in disaggregation algorithms that have previously been struggling with complex usage scenarios. The improvements can directly address the limitations in existing NILM approaches, suggesting that high-frequency monitoring is a promising path toward enhanced disaggregation accuracy for real-time energy management applications. Future work will focus on expanding the dataset to various appliances over diverse environments, also developing more sophisticated processing capabilities to address storage and processing challenges, integrating complementary alternative sensing methods (temperature, humidity…) and implementing optimized, real-time classification methods suitable for practical deployment. Future work will also include enhancing disaggregation capabilities enabled by high-frequency monitoring, which can support advanced energy management applications such as anomaly detection, predictive maintenance, and targeted energy efficiency advising.

## Figures and Tables

**Table 1:** Summary of existing NILM datasets.

Dataset	Year	Description
REDD [6]	2011	Provides both whole-house and circuit-level power measurements from multiple homes.
UK-DALE [7]	2014	Offers long-term periods of measurement across multiple homes and at a higher temporal resolution.
BLUED [8]	2012	Focuses on detecting and labeling individual appliance switching events rather than capturing continuous operation.
AMPds [9]	2013	Provides long-term monitoring, with one-minute resolution for a single house with 21 sub-metered loads.
Our Dataset	2025	Captures a diverse set of electrical features with up to 32 harmonic components, using a scalable, low-cost system. Publicly available and reproducible, enabling community contributions and extensions.

**Table 2:** Measurement parameter range from the ATM90E36A datasheet [20].

Measurement	Range
Voltage	0–655.35 V
Current	0–65.535 A
Voltage rms	0–655.35 V
Current rms	0–65.535 A
Active/Reactive Power	−32.768–+32.767 KW/kvar
Apparent Power	0–+32.767 kVA
Frequency	45.00–65.00 Hz
Power Factor	−1.000–+1.000
Phase Angle	−180°–+180°
THD+N	0.00–99.99%
THD	0.00–399%
Harmonic Component	0.00–399%

**Table 3:** Performance comparison of RF, SVM, and CNN models. Lower values indicate better performance (↓).

Device	Model	MRE (↓)	MAE (↓)	MSE (↓)
Hair Dryer	RF	**0.033**	**0.42**	**24.35**
	SVM	0.701	4.63	1541.69
	CNN	0.162	13.16	2540.1
Iron	RF	0.521	**1.88**	**1141.12**
	SVM	**0.221**	16.46	20,833.26
	CNN	2.559	10.50	6639.73
Electric Water Heater	RF	**0.037**	**0.46**	**13.72**
	SVM	2.104	5.41	4289.31
	CNN	3.247	69.05	67,653.79
Hair Straightener	RF	**0.113**	**0.27**	**1.02**
	SVM	3.426	7.77	198.30
	CNN	0.661	1.73	18.59

## Data Availability

The dataset generated and analyzed during the current study, including metadata, file structures, analysis scripts, and example of code, is publicly available in the GitHub repository: https://github.com/fariddinar/nilm-dataset (accessed on 20 June 2025).

## Abbreviations

The following abbreviations are used in this manuscript:

NILM	Non-Intrusive Load Monitoring
IC	Integrated Circuit
DFT	Discrete Fourier Transform
FFT	Fast Fourier Transform
CT	Current Transformer
SPI	Serial Peripheral Interface
DMA	Direct Memory Access
THD	Total Harmonic Distortion
RMS	Root Mean Square
RF	Random Forest
SVM	Support Vector Machine
CNN	Convolutional Neural Network
MAE	Mean Absolute Error
MRE	Mean Relative Error
MSE	Mean Squared Error
